# The Education of Hospital Officers

**Published:** 1915-02-27

**Authors:** Geo. Watts

**Affiliations:** Secretary. City of London Hospital for Diseases of the Chest.


### THE EDITOR'S LETTER-BOX.

### The Education of Hospital Officers.

*To the Editor of* THE HOSPITAL.

SIR,—In your second leading article of the issue of the 6th instant, you laid down in very decided terms the principles upon which associations or societies of members of any particular profession should be founded. Admitting that the working of an association is made rather more difficult by opening its membership to every person embraced in the profession without any further qualification, it does appear to me that this difficulty is exaggerated out of all proportion in the arguments set forth in the article. I regret that the subject is too large to permit of my asking permission to combat your arguments here, and after discussion it might prove still to be a matter of opinion.

The recent appointment of a gentleman who has not had previous hospital experience to the post of secretary of the Hampstead and North-West London Hospital provides the material for the article referred to, but the statement that "a tendency lately on the part of committees not to promote junior hospital men" to the higher administrative posts has been noticed, has the appearance of being consciously vague in the mind of the writer, and it does not, I consider, accord with the facts. An examination of the matter, will, I believe, show that during the past few years it has been exceptional for such posts to be advertised without the requirement of previous experience, and equally exceptional for other than hospital-trained men to be appointed. In the case of the Hampstead appointment, we must assume that the committee had some particular reason for departing from their own proposed restriction. Whatever the disappointment caused to certain of the candidates in that instance, there is no reason, in my opinion, for dismay in regard to a tendency of committees in this respect.

When the educational scheme of the Incorporated Association of Hospital Officers, which is now in progress, bears fruit, and when it is more generally recognised that the hospital service has assumed a definite character (to which the formation of a pension scheme for hospital officers, of which there is now hope, will contribute), there will, I believe, be little reason to fear that qualified junior officers will fail to receive the reward of promotion.—I am, Sir, yours faithfully,

GEO. WATTS, Secretary.

City of London Hospital for Diseases of the Chest.
February 17, 1915.

[We welcome Mr. Watts' interesting letter as the subject is indeed worth thrashing out. The statement in the article which Mr. Watts criticised may be illustrated by the fact that the Hampstead appointment is the second in almost as many months in which candidates for hospital appointments have been successful from outside the ranks of junior hospital men. The general question of training is discussed in our Notes this week.—ED. THE HOSPITAL.]

